# Emerging paradigms: unmasking the role of oxidative stress in HPV-induced carcinogenesis

**DOI:** 10.1186/s13027-024-00581-8

**Published:** 2024-07-02

**Authors:** Arash Letafati, Zahra Taghiabadi, Negar Zafarian, Roxana Tajdini, Mozhgan Mondeali, Amir Aboofazeli, Silvia Chichiarelli, Luciano Saso, Seyed Mohammad Jazayeri

**Affiliations:** 1 Department of Virology, School of Public Health, Tehran University of Medical Sciences, Tehran, Iran; 2 Research Center for Clinical Virology, Tehran University of Medical Science, Tehran, Iran; 3 Department of Biochemical Sciences “A. Rossi-Fanelli”, Sapienza University of Rome, 00185 Rome, Italy; 4 Department of Physiology and Pharmacology, Vittorio Erspamer”, Sapienza University, Rome, Italy

**Keywords:** Oxidative stress, HPV-mediated carcinogenesis, Smoking, Alcohol, Psychological stress

## Abstract

The contribution of the human papillomavirus (HPV) to cancer is significant but not exclusive, as carcinogenesis involves complex mechanisms, notably oxidative stress. Oxidative stress and HPV can independently cause genome instability and DNA damage, contributing to tumorigenesis. Oxidative stress-induced DNA damage, especially double-strand breaks, aids in the integration of HPV into the host genome and promotes the overexpression of two viral proteins, E6 and E7. Lifestyle factors, including diet, smoking, alcohol, and psychological stress, along with genetic and epigenetic modifications, and viral oncoproteins may influence oxidative stress, impacting the progression of HPV-related cancers. This review highlights various mechanisms in oxidative-induced HPV-mediated carcinogenesis, including altered mitochondrial morphology and function leading to elevated ROS levels, modulation of antioxidant enzymes like Superoxide Dismutase (SOD), Glutathione (GSH), and Glutathione Peroxidase (GPx), induction of chronic inflammatory environments, and activation of specific cell signaling pathways like the Phosphoinositide 3-kinase, Protein kinase B, Mammalian target of rapamycin (PI3K/AKT/mTOR) and the Extracellular signal-regulated kinase (ERK) signaling pathway. The study highlights the significance of comprehending and controlling oxidative stress in preventing and treating cancer. We suggested that incorporating dietary antioxidants and targeting cancer cells through mechanisms involving ROS could be potential interventions to mitigate the impact of oxidative stress on HPV-related malignancies.

## Introduction

The human papillomavirus (HPV), a virus with double-stranded DNA, has a high tendency to infect humans. HPV infections are common and typically clear up within 12–24 months [1]. Nevertheless, a minority of infected individuals may lead to cancer, accounting for nearly 4.5% of global cancer instances [2]. HPV has a particular tropism towards squamous epithelium cells [3]. Chronic infections with high-risk HPV strains, such as HPV-16, HPV-18, HPV-31, and HPV-33, could contribute to the malignancies [4]. HPV plays a major role in cervical, vaginal, vulvar, anal, and penile cancers. Furthermore, HPV is also linked to head and neck cancers (HNC), specifically oral cavity, oropharyngeal, and laryngeal cancer [5]. In 2020, as reported by the World Health Organization, there were 604,000 recent cervical cancer diagnoses and 342,000 associated fatalities [6]. Cancer development is a complex, multistage process involving various cellular and molecular events leading to the converting a normal cell into a malignant neoplastic cell [7]. The oncoproteins E6 and E7 of HPV are responsible for HPV oncogenic properties as they inhibit the tumor suppressors p53 and pRB [1]. However, as an alternative mechanism, simultaneous episomal expression of HPV E2, E4, and E5 can also enhance vulnerability to cancer initiation, particularly in cases of Head and Neck Squamous Cell Carcinoma (HNSCC) [3].

Reactive oxygen species (ROS) are released as a byproduct of regular cellular metabolism and play a critical role in normal cellular signaling [8]. The impact of ROS on a cell is determined not only by their concentration within the cell but also through the equilibrium of ROS and endogenous antioxidants. When this balance is disrupted, oxidative stress (OS) occurs, damaging and altering various intracellular molecules, including DNA, RNA, proteins, and lipids [9]. OS facilitated by ROS might contribute to the development of cancer by changing cell redox balance, activating proinflammatory pathways, damaging DNA and promoting cell proliferation through protein oxidation [10]. Failure to detoxify ROS with antioxidants can cause a surge in OS. This heightened OS can potentially change the activity and structure of vital cellular macromolecules, such as DNA, leading to abnormal cell growth, mutation, and/or chromosome instability. Ultimately, these changes can result in the formation of neoplasms [11]. E6* expression, which is a shorter isoform of HPV E6 oncoprotein can cause elevated levels of ROS, consequently leading to increasing DNA damage. This effect could be attributed to the reduction in antioxidant capacity, as E6* expression diminished antioxidant enzymes such as superoxide dismutase isoform 2 (SOD2) and glutathione peroxidase (GPx) [12]. These findings provide evidence for a sequence of events that begins with the onset of OS, followed by DNA damage, viral integration, and, ultimately, carcinogenesis [13].

OS is gaining attention as a co-factor in HPV-mediated carcinogenesis. This article provides an overview of ROS generation and its biological effects. It also summarizes current knowledge regarding OS's impact on HPV-mediated carcinogenesis, including epigenetic modification, signaling pathways, and inflammatory responses. We will also review factors influencing the transition from HPV infection to carcinogenesis, such as dietary antioxidant components, alcohol consumption, smoking, and psychological stressors in this complex process.

### HPV infection and carcinogenesis

**Fig. 1:**
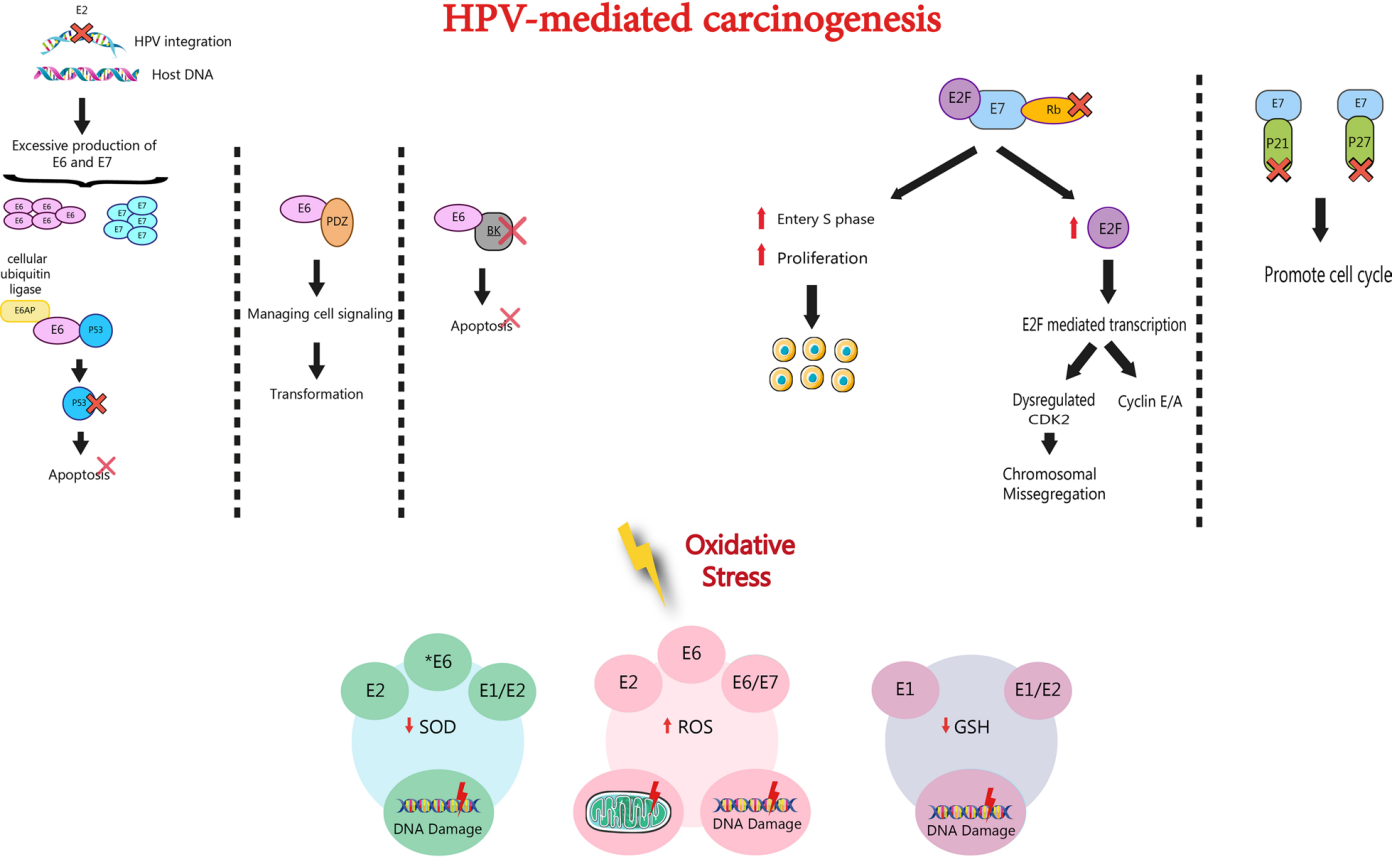
Various processes are involved in HPV-mediated carcinogenesis and the impact of oncoproteins on oxidative stress [21, 22, 52, 53]. In HPV-mediated carcinogenesis, integration of HPV DNA into the host leads to the disruption of E1 and E2 reading frames, resulting in overexpression of E6 and E7 oncogenes. E6 inhibits tumor suppressor protein (p53) and pro-apoptotic protein (BAK), preventing apoptosis and facilitating viral DNA replication. E6 also targets PDZ proteins, enhancing transformative capabilities. E7 interacts with the tumor suppressor (pRb), promoting carcinogenesis by disrupting cell cycle regulation. E7 facilitates E2F-induced transcription by releasing E2F from pRb-E2F complexes, increasing CDK2 activity and centrosome amplification. E7's CD3 domain interacts with proteins like p21 and p27, suppressing their activity and allowing cells to bypass DNA damage-induced cell cycle arrest. These mechanisms collectively create an environment conducive to viral DNA replication and malignant cell transformation [18, 20–23, 25, 26, 28, 54]

HPV infections are usually symptomless and clear up naturally with a robust immune response. However, under specific conditions, like immunosuppression, latent infections can reactivate and, in some instances (especially with HPV types 16 and 18) may progress to cancer [14, 15]. The HPV DNA contains eight open-reading frames, categorized into early (E1, E2, E4, E5, E6, and E7) and late (L1 and L2) regions [16]. The HPV E1 protein is crucial for amplifying the viral episome within host cells, as it is the only enzyme in HPV with ATP-dependent helicase activity, facilitating the replication of the HPV genome [16, 17]. As shown in Fig. 1 in HPV-mediated carcinogenesis, the integration of the viral DNA into the host disrupts the reading frame of E1 and E2, resulting in overexpressed E6 and E7 oncogenes [18]. E2 protein functions as a regulator for the expression of the E6/E7 [19]. E6 interferes with p53 (tumor suppressor) and BAK (pro-apoptotic protein), inhibiting their function and preventing apoptosis, enabling viral DNA replication [18]. Proteasomal degradation of p53 and its inhibition is constituted through E6 binding to the ubiquitin ligase enzyme, referred to as E6 associated protein (E6AP) [20, 21]. Consequently, cells expressing E6 experience a significant reduction in p53 levels, making them prone to the aggregation of chromosomal abnormalities. The primary function of p53 is to protect the integrity of the cell's genome. When DNA damage is present, p53 is crucial in triggering cell-cycle arrest or promoting programmed cell death (apoptosis) [22]. E6 also possesses numerous p53-independent targets that actively contribute to its transformative capabilities. These targets contain a range of crucial factors, such as PDZ proteins that manage cell signaling and adhesion, the p300/CBP transcriptional activators that participate in differentiation regulation and cell cycle control, as well as proteins involved in apoptosis [23]. E6 also triggers the transcription of telomerase reverse transcriptase (TERT), which plays key role in cell immortality [24].

E7, another major HPV oncoprotein, interacts with pRb (tumor suppressor) to develop carcinogenesis [25]. pRb assumes a pivotal function in the cell cycle by regulating the shift of cells from the G1 to the S phase. In the absence of phosphorylation during the early G1 phase, pRb engages with E2F transcription factors and suppresses the transcription of the E2F-regulated genes. However, as the cell moves closer to the S phase of the cell cycle and in late G1, pRb undergoes phosphorylation, releasing E2F. Genes under the control of E2F encompass cell cycle regulators like Cyclin E and Cdc25A, as well as genes responsible for DNA replication [26]. E7 connects with unphosphorylated pRb using the LXCXE motif, facilitating its ubiquitination and subsequent degradation via the proteasome. By disrupting the pRb-E2F complex, E2F is released, triggering E2F-induced transcription. This, in turn, leads to elevated levels of cyclin-dependent kinase-2 (CDK2), as well as cyclins A and E [25]. E7's deregulation of CDK2 function sparks the amplification of centrosomes, a distinct characteristic observed in malignant cells [27]. The E7-dependent inhibition of pRb activity emerges as a pivotal factor in assisting with the shift from one stage to another in the cell cycle during the process of epithelial differentiation. This process creates an ideal environment for viral DNA replication while simultaneously driving malignant cell transformation. E7 could interacting with various cellular proteins, such as the CDK inhibitors p21 and p27. By suppressing their activity, E7 enables cells to evade cell cycle arrest stimulated by DNA damage. Moreover, E7 activates DNA methyltransferase, resulting in an uncontrollable rise in DNA methylation levels. This disruption further interferes with the epigenetic regulation of cellular processes [20, 21, 28].

In addition, the HPV E5 protein could also be involved in the development of carcinogenesis. Research has revealed that the E5 oncoprotein of HPV16 is associated with cervical lesions and may play a role in carcinogenesis by affecting cell proliferation and differentiation as well as apoptosis [29]. Lastly, E4, another HPV protein, was thought to facilitate the release of virions from superficial cell layers by disrupting keratin fibers [30].

### Creation and consequences of oxidative stress

ROS can originate from both endogenous and exogenous sources. Inflammatory cells (eosinophils, neutrophils, and macrophages), mitochondria, and peroxisomes are endogenous origins of ROS [31, 32]. The exogenous origins of ROS are exposure to ionizing radiation (IR), ultraviolet (UV) radiation, biological organisms, pollutants, food, alcohol, and tobacco [33, 34]. OS is characterized by the disproportion between the production of ROS and RNS (reactive nitrogen species) and antioxidants [35]. During the process of oxygen reduction, intermediate products attack to cellular DNA, lipids, and proteins, creating reactive compounds that can subsequently interact with DNA bases [36]. ROS also are involved in the onset, promotion, and advancement phases of tumor growth. The participation of ROS in cancer development is evidenced by the presence of oxidative DNA alteration in tumor tissues. During the promotion stage, cells undergo identical mutations due to the stimulation of cell growth and/or suppression of apoptosis [37]. In this phase, ROS crucially promotes the expansion of mutant cell clones. This is accomplished by temporarily adjusting the activity of genes responsible for either cell growth or cell death [38]. Excessive ROS levels can induce transcription factors like nuclear factor-kappa B (NF-kB), which is accountable for regulating cell growth and oncogenesis [37, 39]. Elevated DNA modification caused by oxidative damage may contribute to genetic instability and potentially significantly impact tumor metastases [36, 37]. OS can potentially trigger damage to the host DNA, which in turn can make it easier for HPV DNA to integrate into infected cells [40].

### Carcinogenesis of HPV through induction of ROS

#### Mitochondrial dysfunction

HPV can lead to carcinogenesis by disrupting mitochondria, a cellular powerhouse responsible for metabolism, cell growth, cell survival, apoptosis, and generating superoxide anions as byproducts during their oxidative phosphorylation (OXPHOS) in energy production process [40–42]. A study found that E1 and E4 proteins of HPV16 can attach to mitochondria, causing them to detach from microtubules. This results in the clustering of mitochondria near the cell's nucleus, leading to a decline in mitochondrial membrane potential and the initiation of programmed cell death [41]. The localization of HPV18 E2 within the mitochondrial membrane leads to morphological changes of cristae, resulting in an amplified release of ROS from mitochondria. ROS promotes an increase in glycolysis in cellular respiration, shifting away from OXPHOS, which may contribute to tumorigenesis by triggering the Warburg effect [42]. Additionally, Gregorio et al. demonstrated that HR-HPV18 E2 amplifies the production of ROS and causes a diminish in cellular glutathione (GSH) levels. Moreover, the combined expression of E2 and E1 proteins from HR-HPV18 triggers even higher levels of OS and DNA damage compared to E2 alone. This finding implies that E1 and E2 collaborate to enhance OS during the replicative cycle of HR-HPV, potentially leading to apoptosis [43].

The HR-HPV16 E2 protein engages with a mitochondrial protein (gC1qR: a receptor responsible for binding to the globular heads of C1q) that plays a role in inflammation and immune response. This interaction leads to mitochondrial abnormal function, elevated ROS generation, and triggers apoptosis through the activation of the p38 MAPK/c-jun N-terminal kinases (JNK) signaling cascade in cervical cancer cells [44]. Villota et al. observed a distinctive expression pattern in human mitochondrial noncoding ribonucleic acids (ncRNAs) among normal and HPV-infected tumor cells. Specifically, HPV E2 was found to suppress the expression of ASncmtRNAs (antisense noncoding mitochondrial tRNA). Furthermore, the oncoproteins E6 and E7 were found to stimulate SncmtRNA-2 expression, thereby promoting oncogenesis initiated by HR-HPV [45]. Similarly, in another study, the HR-HPV18 E2 protein appears to contribute to the progression of cervical cancer by suppressing the expression of ASncmtRNA. ASncmtRNA expression is upregulated in normal proliferating cells but diminished in tumor cells [46]. Recent studies revealed that HR-HPV16/18 E6 significantly impacts mitochondrial function in a model of HNSCC. These oncoproteins enhanced mitochondrial metabolism by promoting cellular respiration and the activity of mitochondrial complexes (I-V). Although there was an increase in mitochondrial function, there was no increase in ATP production. Instead, this heightened activity led to an elevated parameter of leak mitochondrial respiration, resulting in the formation of ROS within both the mitochondria and the whole cells. Consequently, this OS caused DNA damage [47]. Evans et al. revealed that the elevated expression of HPV16 E6* led to mitochondrial impairment, increased generation of ROS, and reduced GSH levels. These effects were found to be associated with the modification of pathways related to mitochondrial malfunction in cervical cancer cells [48]. The research proved that the oncoprotein E7 from HR-HPV16 stimulated both the expression and activity of catalase by reducing the production of ROS and providing a safeguarding effect against OS; the presence of E7 from HR-HPV16 oncoprotein was observed to prevent apoptosis through the mitochondrial pathway in HaCaT keratinocytes. (HaCaT cells are immortalized human keratinocytes widely utilized for studying epidermal homeostasis and its pathophysiology) [43, 49, 50]. In addition, in HPV + head and neck cancer (HNC), E6 and E7 induce OS through NADPH oxidase (NOX) enzymes, leading to DNA damage, oxidative base lesions, and also DNA SSB chromosomal irregularities [51].

HPV oncoproteins disrupt mitochondrial function, leading to OS and DNA damage, promoting carcinogenesis. The intricate interplay between HPV and mitochondrial processes highlights potential therapeutic targets for HPV-associated cancers. Understanding these mechanisms is crucial for developing targeted interventions to counteract HPV-induced mitochondrial dysfunction and halt tumorigenesis.

#### Inflammatory responses

The inflammatory reaction begins when the immune system receptors detect the presence of infecting pathogens or any cellular damage [55]. The immune system receptors known as pattern recognition receptors (PRRs) are specific transmembrane receptors found in immune system cells. These receptors recognize conserved molecular components in infecting microorganisms and internal injuries, which are identified as pathogen-associated molecular patterns (PAMPs) and danger-associated molecular patterns (DAMPs), respectively [56, 57]. Several PRRs have a selective ability to detect PAMPs, such as Toll-like receptors (TLRs) and also retinoic acid-inducible gene 1 (RIG 1)-like receptors (RLRs) [58]. The RLRs were shown to recognize viral-infected cells through the detection of cytoplasmic dsRNAs [59]. TLRs are present in both cell membranes (TLR1, 2, 4, 5, and 6) and endosomes (TLR3 and 9) and are pivotal in host cell recognition and antiviral immunity [60]. Activation of these PRRs induces the activity of NF-κB as a major transcription factor involved in inflammation and OS responses. NF-κB triggers the production of inflammatory cytokines and accelerates immune responses by modifying the vascular endothelial permeability and facilitating the recruitment of macrophages and neutrophils as well as natural killer (NK) cells in the infection location [61, 62]. ROS triggered NF-κB inducing inflammatory cytokines. Inflammation, in turn, can cause OS through neutrophil-generated ROS [63]. The NF-κB also can impact the level of ROS by modulating the expression of SOD1/SOD2, Glutathione S-transferase pi (GST-pi) and Gpx1 [64]. Acute inflammation resolves after removing the infecting pathogens and repairing damaged tissues, but if these disorders are not repaired quickly, inflammation will become chronic. Consistently, persistent viral infection promotes chronic inflammation, accumulating of prostaglandins, leukotrienes, and ROS that can increase the risk of DNA damage, mutation, and carcinogenesis [65, 66].

HPV has developed numerous strategies to circumvent or inhibit immune responses by regulating the expression of the inherent immune system components and also neutralizing the acquired immune responses, HPV persistent infection and progress of multiple malignancies, including cervical, vulvar, and vaginal cancers, occur [67, 68]. During replication of HPV, the virus can trigger chronic inflammatory microenvironment via IL-1β, IL-6, and also attracting immune cells to the lesion area [69]. In HPV infection, TLR9 induces the NF-κB-dependent production of type-1 IFNs by promoting several nuclear transcription factors, such as interferon regulatory factor 3 (IRF3) and IRF7 [70]. Recent studies on the antiviral effects of IRFs in HPV indicate that upregulation of these regulatory factors enhances the expression of IFNs and induces the immune clearance of HPV [71, 72]. Further results indicate that the type-1 IFNs enhance the nuclear translocation of the ISGF3 complex comprising STAT1, STAT2, and IRF3 [73]. The ISGF3 complex inhibits viral replication and decreases the proliferation of viral infected cells by facilitating the expression of various antiviral agents, including 2′,5′-oligoadenylate synthase (OAS), and protein kinase R (PKR) [74, 75]. To discern the molecular process engaged in the viral immune escape, it has been shown that infection with HPV16 inhibits the expression of TLR9 by E6 and E7, resulting in the downregulation of inflammatory cytokines like type-1 IFNs and inhibition of immune responses [76]. In addition, the study on HPV E6 and E7 in cervical cancer found that they regulate the expression of CXCL1, CXCL2, and CXCL3, as well as CXCL8 chemokines [77]. Therefore, the immune system targets viral oncogenes expression through OS; however, excessive OS may facilitate HPV DNA integration [78]. Williams et al. suggested that inflammation, through the production of ROS, can trigger DNA strand breaks in both host and virus, facilitating the integration of the HPV genome into the host cell chromatin [40]. In a study examining the impact of HPV infection on inflammation and OS, elevated levels of cytokines (IFN-γ, IL-1β, and IL-6), lipid peroxidation, and 8-hydroxydeoxyguanosine (8-OHdG) were observed alongside decreased total antioxidant capacity. 8-OHdG functioned as an indicator of OS, signifying the presence of DNA oxidation [79]. Similarly, elevated levels of cytokines were accompanied by OS marker (lipid peroxidation) and decreased levels of catalase and superoxide dismutase antioxidant enzymes in high-risk HPV patients. These results imply that HPV infection triggers a persistent inflammatory reaction and plays a role in generating an oxidative environment [80].

Inflammation initiated by HPV triggers NF-κB-mediated cytokine production and immune cell recruitment, leading to chronic inflammation and oxidative stress. HPV employs strategies to evade immune responses, promoting viral persistence and progression of malignancies, including cervical cancer. Excessive oxidative stress may facilitate HPV DNA integration, exacerbating inflammation and contributing to carcinogenesis.

#### The interplay between HPV infection and cellular signaling pathways

In HPV-related cancers, multiple oncogenic signaling pathways are involved in metabolism, differentiation, and cell proliferation [53]. As mention earlier, persistent infection of HPV is essential to induce cancer. It has been documented that the upregulation of HPV E6, and E7 induces diverse cellular signaling cascades implicated in cells becoming cancerous and immune escape [81]. It has been shown that HPV oncoproteins induce cell cycle progression by inhibiting several tumor suppressors, including p53, PDZ, and retinoblastoma, resulting in tumor initiation and development [81, 82]. PI3K/AKT/mTOR pathway, as one of the central signaling routes regulated in HPV‐related cancers, was implicated in regulating cell proliferation, migration, invasion, and angiogenesis [83]. The over-activation of PI3K/AKT signaling was reported in cervical squamous cell carcinomas like cervical cancer [84]. PI3K signaling was also reported to be activated by HR‐HPV16 E7 in oral cancer. Upregulation of AKT promoted the expression of *Pirin* and subsequent activation of nuclear factor erythroid 2‐related factor 2 (Nrf2) and the NF-κB signaling cascade, protecting cells from OS effects on viability [53]. *Pirin* is an OS sensor that is upregulated in various cancers and acts as a potent transcriptional activator of NF-κB, resulting in OS, cell migration, and metastasis [85, 86]. Moreover, it has been shown that HR‐HPV16 downregulates the mTOR inhibitor tuberous sclerosis complex 2 (TSC2) and promotes the activity of mTOR complex 1 (mTORC1), resulting in cell growth, metabolism, and proliferation [87]. Another signaling route that HPV promoted was the extracellular signal-regulated protein kinase (ERK) pathway that engaged in cell growth, EMT, and metastasis [88]. Upregulation of ERK signaling induces various EMT-related proteins, including Snail and Twist, and matrix metalloproteins (MMPs) implicated in cellular migration, invasion, and the spread of cancer cells [89]. Carrillo et al. examined the oncogenic mechanism of HPV in oral and cervical cancer. Their findings indicate that HR‐HPV16 E7 induces the activity of the EGFR/MEK/ERK axis and upregulates the OS sensor Pirin protein, resulting in cell motility and EMT [85]. Further studies on the relationship between ROS levels and ERK signaling in HPV16-positive cervical cancer revealed that decreased ROS levels cause reduced activity of the ERK signaling pathway. Mechanistically, ROS maintains ERK signaling activity by inhibiting ERK phosphatase dual‐specific phosphatase 3 (DUSP3) resulting in increased cell migration and EMT [53, 90].

The wnt/β-catenin pathway is another signaling route that is upregulated in HPV-related cancers. The Wnt/β-catenin axis significantly influences cell proliferation, migration, and differentiation [91–93]. In HPV-induced cancers, the Wnt signaling is activated by HPV E6 and E7 oncoproteins through inhibiting various regulatory factors such as p53, human telomerase reverse transcriptase (hTERT), and protein phosphatase 2A (PP2A) [94]. Further studies on the regulatory effects of HPV on Wnt/β-catenin signaling indicate that HPV E6 and E7 oncoproteins stabilize β-catenin proteins in the cytoplasm by inhibiting the degradation complex of β-catenin and PP2A in cancer cells [95]. Additionally, the E6 facilitates the nuclear translocation of β-catenin and induces the expression of Wnt signaling downstream target genes like cyclin D1 and c-MYC, leading to cell growth and proliferation [96]. Recent studies on the Wnt signaling effects on reducing immune responses against HPV-induced cancer cells indicate that c-MYC upregulates PDL1 on the surface of cancer cells. Interaction between PDL1 in cancer cells and PD1 in T cells causes apoptosis and suppresses immune responses against tumor cells [97]. Therefore, the downregulation of Wnt/β-catenin signaling and PDL-1 probebly have therapeutic effect against HPV-induced cancers.

HPV oncoproteins intricately manipulate multiple signaling pathways to drive tumorigenesis and evade immune responses, underscoring the significance of targeted therapeutic interventions in HPV-associated cancers.

### DNA damage

ROS is among the most prevalent causes of DNA damage produced during inflammatory processes, cellular metabolism, infections, and chemical or mechanical stress [98]. ROS generates around 10,000 modifications in the DNA bases, resulting in dsDNA/ssDNA breaks, DNA intrastrand adducts, and cross-links to other molecules [40]. In cervical cancer, the levels of 8-nitroguanine and 8-oxodG, which are two main forms of oxidative DNA damage, were linked to higher grade of cervical intraepithelial neoplasia (CIN) [99]. In addition, HR-HPV infection cases exhibited higher serum status of malondialdehyde (MDA), a serum marker of lipid peroxidation, compared to uninfected controls [100]. MDA, which is formed through the process of lipid peroxidation, can cause DNA impairment by creating exocyclic adducts [101].

**Fig. 2:**
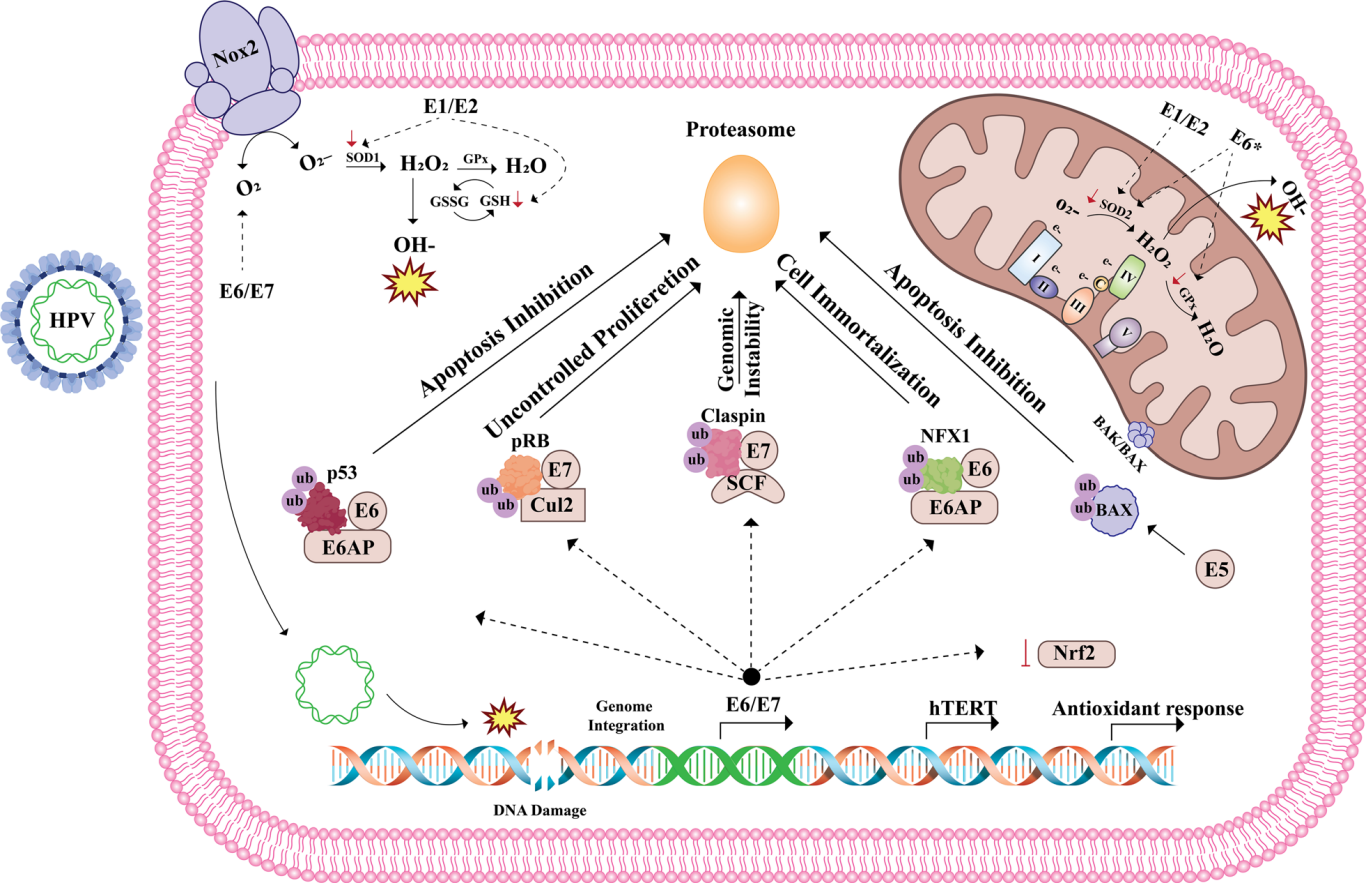
Mechanism of HPV-mediated carcinogenesis following oxidative stress and DNA damage. HPV oncoproteins regulate the redox system in different ways. E1/E2 co-expression induces ROS production by decreasing the GSH/GSSG rate (reduced vs oxidized form of GSH) and the activity of SOD1/2 [43]. E6*, an isoform of E6, diminishes the levels of GPx and SOD2 proteins, thereby raising OS levels [12]. E6/E7 oncogenes lead to an increase in ROS levels by inhibiting Nrf2 activity, one of the critical transcription factors contributing to the antioxidant response, and activating Nox2 oxidase [51, 112]. ROS-induced DNA damage, especially DSB facilitates the integration of HPV into the human genome and promotes the overexpression of E6 and E7. E6 oncoprotein induces the apoptosis inhibition and immortalization of host cells through the degradation of p53 and NFX1 proteins. NFX1 is a transcriptional repressor binding to the TERT promoter and represses its expression [113, 114]. On the other hand, E7 oncoprotein promotes uncontrolled proliferation and genomic instability by degradation of pRB and claspin, a primary regulator of the ATR repair pathway [108, 115]. E5 is also capable of inhibiting apoptosis by proteasome-mediated degradation of proapoptotic BAX [116]. Glutathione (GSH), glutathione disulfide (GSSG); superoxide dismutase (SOD); glutathione peroxidase (GPx); nuclear factor erythroid 2–related factor 2 (Nrf2); NADPH oxidase 2 (Nox2); nuclear transcription factor X box-binding protein 1 (NFX1); Telomerase reverse transcriptase (TERT)

Generally, the cells activate the DNA damage response (DDR) as a result of DNA impairment [102]. This process entails a complex gene network responsible for sensing and repairing errors, ensuring genome integrity and cell survival [103]. Interestingly, HPV-infected cells adapt to OS situations by inhibiting OS-induced apoptosis and modulating antioxidant activity. These mechanisms are mediated by HPV oncogenes that have a significant impact on bypassing DDR pathways [104]. E6 protein can create a ternary complex with p53 and ubiquitin ligase (E6AP), causing to the ubiquitination and proteasomal degradation of p53 [105]. This protein is needed to detect base excision repair machinery (BER), and its degradation induces apoptosis and disturbance of cell cycle regulation [106]. E6 can also function at the mRNA level to trigger the expression of human telomerase reverse transcriptase (hTERT), contributing to maintaining telomeres length and immortalization [107]. Williams et al. showed that E6* isoform, a truncated form of E6, raises ROS levels and DNA damage by lowering the SOD 2 and GPx 1/2 antioxidant proteins [12]. The attachment of E7 to pRb inhibits its interaction with the E2F transcription factors, eliciting the transcription of S-phase related genes and an uncontrolled proliferation [21]. E7 protein also interacts with the ATR DNA damage pathway to increase the degradation of claspin, a crucial controller of the ATR/CHK1 signaling axis, thereby reducing the DNA damage checkpoint control in the G2 phase [108]. Liu et al. proved that HPV infection could shift cancer cells from homologous-recombination DNA double-strand-break (HR) repair to alternative end-joining (altEJ) by impairing TGFβ signaling. This repair pathway acts in an error-prone manner, probably promoting chromosomal aberrations [109]. Thus, the HPV employs the DDR machinery to trigger its replication in the presence of DNA damage, particularly DS breaks of the host genome, allowing multiple viral integrations [110]. The integration occurrence results in the elimination of the repressive role of E2 and persistent overexpression of the E6 and E7, driving cellular functions toward a carcinogenic process [111]. Figure 2 displays the mechanism of HPV-mediated carcinogenesis following OS and DNA damage.

Consequently, ROS-induced DNA damage, mediated by HPV infection, underscores the intricate interplay between oxidative stress and genomic instability, promoting carcinogenesis via dysregulation of DNA repair pathways and evasion of cell cycle checkpoints. Targeting these mechanisms presents potential avenues for therapeutic intervention in HPV-associated cancers.

#### Epigenetic modifications

Epigenetic modifications are closely intertwined with gene expression, as they encompass changes to gene function that occur independently in alterations in the DNA sequence [117]. These modifications could regulate the activation or deactivation of genes [118]. The diverse range of mechanisms involved in epigenetic changes encompass DNA methylation, histone alterations, and post-transcriptional modifications facilitated by non-coding RNAs such as miRNA [119]. Many studies have highlighted how epigenetic abnormality in host and viral DNA, resulting from viral integration into the human genome, drives HPV's progression towards carcinogenesis. These changes affect the expression of genes like tumor suppressors and E6/E7 oncogene, as well as the DNA repair process [120–122].

Histone modifications, including acetylation via histone acetyltransferases (HAT) and deacetylases (HDAC), ubiquitination, methylation, and phosphorylation, serve as factors in remodeling chromatin, tied to epithelial differentiation [123, 124]. Oxidative genome damage in HPV can lead to epigenetic changes associated with tumorigenesis [110]. In the presence of OS, HPV undergoes a cascade of epigenetic alterations to regulate cellular redox equilibrium and modulate cell proliferation. In mouse cervical tumors, lactate dehydrogenase A (LDHA), as an enzyme involved in energy metabolism, is abnormally translocated to the cell nucleus due to the increase of intracellular ROS caused by HPV-16 E7. Subsequently, LDHA generates α-hydroxybutyrate and initiates histone modification by methylating H3K79 through DOT1L as a disruptor of telomeric silencing 1-like [125].

The interplay between epigenetic regulation via histone methyltransferase and the presence of immune checkpoint molecules, specifically Tim-3 and galectin-9, holds significance in cervical cancer cases, particularly those linked to HPV-18 infection. These molecules are involved in immune evasion strategies [126]. Within this context, Enhancer of Zeste Homolog 2 (EZH2) mRNA emerges as a histone methyltransferase gene responsible for adding methyl groups to histone proteins and raised in cervical cancer [127]. Notably, two transcription factors, E2F-1 and FOXM1, are activated by HPV18 E6 and E7 proteins, leading to an augmentation in EZH2 activity and subsequent suppression of DNA methyltransferase 3A (DNMT3A) expression [126].

Topchu et al. show the impact of reduced function of histone methyltransferase, such as NSD1 and NSD2, accountable for catalyzing the dimethylation of lysine 36 on histone H3 (H3K36me2) in HNSCC cells [128]. Histone modifications like H3K36me2 are vital for controlling the structure and function of chromatin, which, in turn, affects DNA repair and transcription processes [129]. An investigation on HNSCC revealed a decrease in H3K36me2 levels, a substantial diminishment in cell growth, and heightened apoptosis among HNSCC cells. Additionally, the study proved that the downregulation of NSD1 led to a decrease in E2F transcription factor gene expression [128]. Two histone acetyltransferases, Tip60, and p300, had distinct effects on histone modifications, particularly on histone H3K9, in the LCR of HPV-18. The LCR of HPV-18 exhibited a bivalent chromatin, bearing both the active histone modification of H3K9 acetylation and the inhibitory histone H3K9 trimethylation. In HeLa cells, the equilibrium between these marks was intricately controlled by Tip60 and p300, where Tip60 serves as a co-repressor, while p300 was responsible for the activation of E6 and E7 [130].

DNA methylation has been observed in both the LCR, a non-coding region controlling viral gene expression, and the E2 gene [131, 132]. Due to methylation in the LCR region, the E2 becomes unable to bind to this segment of the HPV promoter. Consequently, the regulatory influence of E2 on the E6 and E7 oncoproteins is no longer effective [133]. In patients infected with HPV-16, a positive relationship was noted in the methylation of the L1 gene within CpG regions and the presence of cervical cancer. This methylation was found to increase significantly as the severity of lesions increased [121].

Methylation in host DNA, especially in genes pivotal for cell cycle regulation, apoptosis, DNA repair, and the Wnt signaling cascade, occurs during HPV-mediated carcinogenesis [110]. Khan et al. linked the downregulated expression of the p53 gene in cancer patients to its epigenetic changes, including hypermethylation and deacetylation, shedding light on the role of these modifications in the progression of cervical cancer [134]. Further studies have established a connection between methylation in six host genes (GHSR, SST, ZIC1, ASCL1, LHX8, and ST6GALNAC5) and cervical cancer [135, 136]. Van Keer et al. stated that identifying these hypermethylation of host DNA in urine samples could serve as a non-invasive diagnostic method for low and high-grade cervical diseases [137].

Noncoding RNAs, like microRNAs (miRNAs) and small interfering RNAs (siRNAs), exert their function by interacting with the 3'-UTR (3' untranslated region) of target gene mRNA molecules to the modulation of gene expression [25]. OS can downregulate antiviral genes and miRNAs by affecting transcription factors, thus negatively influencing their expression [138, 139]. miR-34a affects thioredoxin reductase 2 (Trxrd2), which is vital for redox regulation and cancer progression. HPV's E6 degrades p53, suppressing miR-34a, leading to Trxrd2 activity. miR-34a also targets uncoupling protein 2 (UCP2), a ROS regulator. Silencing UCP2 increases ROS, and miR-34a downregulates UCP2, elevating ROS levels. miR-34a lowers SIRT1 levels, enhancing cell sensitivity to apoptosis caused by OS. During HPV infection, E6's inactivation of p53 prevents miR-34a from decreasing, thus allowing SIRT1 to remain functional [139, 140]. Gregorio et al. examined the participate of HPV oncoproteins in cellular redox regulation. They reported that the E6 oncoprotein of HR-HPV led to a diminish in cellular glutathione (GSH) levels, a rise in ROS, and a reduction in catalase levels and activity. The decrease in catalase activity suggests impaired antioxidant defence, which may be linked to epigenetic changes, including miRNA modulation, driven by the E6 oncoprotein [43]. A study investigated the role of miR-206 and its influence on G6PD in cervical cancer [141]. The Glucose-6-phosphate dehydrogenase, or G6PD, produces NADPH, which is vital for preserving decreased glutathione (GSH). GSH, in turn, acts as an antioxidant that neutralizes ROS, thereby safeguarding cancer cells from oxidative harm [142]. A study demonstrated that decreased miRNA-206 expression is indeed connected to tumorigenesis and increased cell proliferation in cervical cancer. Cui et al. reported that in cervical cancer, miR-206 exhibits a specific binding affinity for the 3' untranslated region (UTR) of G6PD. Therefore, miR-206 was found to be downregulated, while G6PD exhibited an increase in expression [141].

Consequently, Epigenetic modifications, encompassing DNA methylation, histone alterations, and non-coding RNA interactions, intricately regulate gene expression, playing a pivotal role in HPV-induced carcinogenesis by modulating oncogene expression, DNA repair processes, and immune evasion strategies. Targeting these epigenetic mechanisms holds promise for developing novel therapeutic interventions in HPV-associated cancers, offering potential avenues for precision medicine approaches.

### Exogenous sources of OS and interaction with HPV

#### Smoking and HPV-induced carcinogenesis

Cigarette smoking with phenolic substances, quinones, and heavy metals as well as free radicals, acts, as a source of ROS, promote OS, which in turn triggers the inflammation response and probably promotes carcinogenesis [143]. Smoking can boost the risk of developing HPV-related cancers, particularly HNSCC, CIN, and cervical cancer [144, 145]. A systematic study documented that individual who smoked had 1.67 times (95% CI = 1.37–2.04) higher likelihood of developing CIN and 2.65 times (95% CI = 1.81–3.88) higher likelihood of developing cervical cancer compared to non-smokers [144]. Moreover, in a study involving 1,473 women, it was discovered that smoking heightened the chance of high-risk HPV infection, and the intensity of smoking was linked to this increase [146]. Current smokers with high-risk HPV types are less likely to clear the infection compared to non-smokers, heightened risk of persistent HPV infection associated with tobacco use [147]. However, smoking was not found to be associated with an elevated likelihood of E7 protein positivity among women who were HR-HPV positive [146].

Smoking can influence HPV virus infectivity through multiple mechanisms, including its impact on virus replication, the heightened produce of E6 and E7 oncogenes, and alterations in immune responses against HPV. Furthermore, smoking can contribute to the advancement of HPV infection towards carcinogenesis by promoting DNA damage and modifying the expression of cellular genes [148]. The HPV protein's dependence on DNA damage responses in infected cells to facilitate the replication of its genome may represent a joining between smoking-induced DNA damage and HPV pathogenesis [149]. The study exposed HPV16-transformed cervical cells to cigarette smoke condensate (CSC) for 72 h. The results showed that exposure to smoking and free radicals led to a dose-dependent increase in DNA damage, affecting both SSBs and DSBs [150]. Further study by Carrillo et al. revealed an elevation in SOD2 status and DNA damage when oral cells, which expressed HPV16 E6 and E7, were subjected to tobacco smoke [151]. SOD2 is an enzyme that involved in defending cells against OS by converting superoxide radicals into less harmful molecules [152]. Moreover, the E6 oncoprotein could increasing SOD2 levels independently of ATM and the PI3K/AKT pathway [151]. Lee et al. reported that the SOD2 gene exhibited significantly higher expression levels in HNSCC patients with a greater in heavy smokers. The SOD2 gene's expression was connected to a reduction in the peroxisome proliferator-activated receptors (PPARs) signaling pathway [153], which plays a role in regulating inflammation [154]. In an in vitro study, Shishodia et al. employed 4-Nitroquinoline N-oxide (4NQO), a known mutagen that mimics the effects of smoking and generates oxygen free radicals, to induce DNA damage in HPV-positive cells. They documented an elevation in double-strand breaks (DSBs) and the activation of homologous recombination (HR) DNA repair proteins like BRCA1 and Rad51 within HPV-positive cells [155].

Exposure to heavy metals from smoking, like cadmium and lead, may induce OS by disrupting cellular thiol buffering systems, depleting antioxidants (e.g., GSH, TXN, PRDX), and generating ROS [63, 156]. Moreover, increased serum chromium in smokers contributes to the production of O2^·−^, H_2_O_2_, and OH [63, 157]. Benzo[a]pyrene (BaP) in Tobacco exerts a direct influence on HPV viral replication by stimulating the MAPK/ERK pathway and subsequently activating CDK1 [98]. Kashyap et al. showed that benzo[a]pyrene (B[a]P) of cigarette smoke resulted in alterations in the expression of E6 and E7. The impact of these risk factors not only enhances clonogenesis and invasion of cervical cancer cells but also induces inflammation by affecting TNF-α and NF-kB signaling, resulting in the modulation of IL-6 and the activation of vascular endothelial growth factor (VEGFA) [158]. Therefore, aside from the DNA damage resulting from the promotion of OS due to tobacco, smoking initiates pathways influenced by ROS, connecting to RAS/MAPK, NF-κB, and AP-1, contributing to inflammation, programmed cell death, proliferation, and cell specialization. [159, 160]. Encounter with tobacco carcinogen 4-(methylnitrosamino)-1-[3-pyridyl]-1-butanone (NNK) was influential on gene expression and cellular transformation in HPV-16-immortalized human cervical cells [161]. Moreover, exposing HPV18 to NNK showed an influence on the creation of pyridyloxybutylated DNA adducts, furthering the process of neoplastic transformation in esophageal squamous epithelial cells [162]. Smoking has been associated with a potential weakening of the immune system in the cervix. This weakening is perhaps manifested through a decrease in Langerhans cells and CD4 lymphocytes [163]. The decreased clearance of high-risk HPV infection in people currently smoking compared to non-smokers, indicating an elevated risk of infection persistence linked to tobacco use, is more likely due to the impression of smoking on the immune system. In this situation, host immunity plays a role in the persistence of infection, which is an important risk factor for emerging cervical cancer [147]. Within a research investigation of women with HPV16 DNA, smokers were more likely to lack HPV16-specific antibodies, which reinforces earlier findings that smoking could elevate the likelihood of enduring infection and long-term cancer susceptibility [164]. Smoking-induced OS activates inflammation and initiates ROS production. This dual process, intended to combat damage and pathogens, can exacerbate OS, potentially contributing to cancer development [143].

In a cross-sectional study involving 3,833 participants, the connection between contact with tobacco smoke and high-risk HPV infection was examined, revealing an odds ratio of 1.32 [165]. Based on the preceding study and numerous investigations demonstrating the link between smoking and HPV infection [146, 150, 165, 166], few studies have contradicted this association [167]. For instance, a systematic study stated that smokers in the HPV (+) the group had a lower likelihood of developing HNSCC compared to those in the HPV (−) group, implying a limited role of smoking in HPV-positive HNSCC cancer [168]. The reporting inconsistency could be because smoking status was determined either through self-reports by the subjects or by identifying the presence of specific cigarette metabolites [165].

Smoking exacerbates HPV-induced carcinogenesis by promoting DNA damage, altering immune responses, and enhancing HPV infectivity through oxidative stress. While strongly linked to increased cancer risk, the interaction between smoking, HPV infection, and cancer development warrants further research due to some conflicting findings.

### Alcohol and HPV-induced carcinogenesis

Alcoholic beverage consumption, recognized as a group 1 carcinogen risk factor by the International Agency for Research on Cancer (IARC) [169], contributed to 4.1% of all cancer cases in 2022, including oropharynx, colon, liver, and breast cancer [170]. A multivariate mendelian randomization examination revealed an independent role of alcohol in oral and oropharyngeal cancer with an odds ratio of 2.1 [171]. Alcohol and smoking together heighten squamous cell carcinoma risk by causing ongoing irritation and inflammation of the esophageal mucosa. This combination can also trigger changes in oncogenes like RAS mutations, which encode p21, suppress tumor suppressor genes, and cause DNA damage, ultimately leading to esophageal squamous cell carcinoma (ESCC) [172]. Notably, KRAS mutations are connected with HPV positivity in head and neck cancer and can contribute to its carcinogenesis [173]. Moreover, alcohol in aggregation with smoking and HPV infection, has been established as a contributing factor in the advancement of HNSCC [174]. This association with head and neck cancers may be attributed to free radical damage, interference with DNA repair mechanisms, and the presence of carcinogenic compounds like N-nitrosodiethylamine found in beer [171]. HPV infection may trigger specific anti-apoptotic, proliferative, and malignant cellular reactions, which can be further intensified when combined with the impact of alcohol and tobacco [172]. Alcohol consumption may notably link to a heightened risk of high-risk HPV infection. A study conducted on HNSCC patients reported a noteworthy correlation between alcohol consumption and high-risk HPV infections [175]. Furthermore, alcohol drinker was associated with a higher likelihood of persistent high-risk HPV infection among women with low-grade squamous lesions or lower cytological findings after 1 to 2 years of follow-up, compared to non-drinkers [176]. Similarly, in a cohort study involving 1243 participants, alcohol consumption was linked to over twice the odds (OR = 2.18, 95% CI 1.22–3.89) of developing CIN1 [177]. Therefore, alcohol could play a role in the early stages of HPV infection and carcinogenesis [176]. Moreover, according to Lai and his colleagues, HPV-positive patients with oropharyngeal squamous cell carcinoma (OPSCC) had a worse disease-free survival (DFS) outcome if they drank alcohol, causing 26.1% elevated risk of disease relapse, which consequently reduced the positive effect of HPV status on their prognosis [178]. A prospective study revealed that current drinkers and those with a drinking history of 5 years or more had a enhanced risk of 2-year HR-HPV persistence, which potentially contributed to cervical cancer. Variations in the levels of amines and amine oxidases in cervical mucus can lead to a diverse host response to viral infections, potentially influenced by cervical ROS [179].

Drinking alcohol leads to a low-oxygen environment with higher levels of ROS, primarily caused by the action of CYP2E1, leading to damage to DNA. This OS and the breakdown of acetaldehyde can result in the creation of DNA adducts and may be associated with epigenetic modifications of DNA, like methylation [180]. The breakdown of ethanol via alcohol dehydrogenase (ADH) results in the creation of acetaldehyde and the onset of OS, which, when combined, synergistically contribute to an increase in HPV virus load. Consequently, this contributes to both the initiation and development of cervical tumorigenesis [181]. OS can contribute to HPV- mediated carcinogenesis by increasing the risk of infection and establishment of a long-lasting infection, as well as influencing the integration of the HPV DNA into the host genome [98]. Prolonged and excessive alcohol consumption activates the hepatic microsomal ethanol-oxidizing system, including the cytochrome P-450 (CYP) enzyme, which metabolizes alcohol and generates ROS like hydroxyethyl radical, ethoxy radical, acetyl radical, and others [182]. Ethanol-induced CYP2E1 expression generates ROS, leading to OS and DNA damage. This can result in genome mutations and cell immortality as well as clonal expansion, ultimately contributing to cancer development. ROS can oxidize DNA, causing alterations like oxidized bases, SSB damage, and DNA adducts [180]. Additionally, consuming alcohol has the potential to damage mitochondria and trigger OS [183]. Ethanol may have the capacity to elevate the activity of nicotinamide adenine dinucleotide phosphate oxidase (NOX) or NADPH Oxidase enzymes, which, in turn, can lead to the formation of ROS [180].

Acetaldehyde is considered a ROS that can be formed as a outcome of the metabolism of ethyl alcohol (ethanol) through the action of the ADH enzyme [169, 184]. Acetaldehyde, present in premalignant as well as malignant lesions, induces DNA damage by forming bulky adducts and inhibiting DNA repair mechanisms like O6-methylguanine transferase (MGMT) [184, 185]. It also enhances cellular permeability, causes DNA damage through the generation of bulky adducts like N2-acetaldehyde deoxyguanine, alters the activity of enzyme convertases, and disrupts cellular oxidative balance by binding to glutathione reductase, resulting in lipid peroxidation and the formation of hydroxyl radicals [184]. Alcohol dehydrogenase (ADH) first converts ethanol to acetaldehyde, which is then further metabolized by aldehyde dehydrogenases (ALDH) to acetate [186]. ALDH2 can play a role in tumorigenesis and cancer progression mechanisms [187]. Gui et al. explored the joining between p53 and ALDH in cancer development and discovered greater diversity in ALDH isoform expression in p53-inactivated (p53WT) cases due to the HPV16 E6 oncogene in HNSCC patients. This diversity in ALDH isoforms influenced the prognosis of HNSCC in p53WT cases but not in p53HRmut cases [188].

Unlike studies suggesting a potential link between alcohol consumption and high-risk HPV infection in cancer [175–178], recently several studies have presented contrasting findings [189, 190]. Auguste et al. investigated the role of tobacco and alcohol in HNSCC and their interaction with HPV infection. They found that the effects of tobacco and alcohol, both individually and when combined, were less significant in HPV-positive HNSCC patients compared to HPV-negative cases [189]. Similarly, A systematic study found that the risk of progression of OPSCC was lower among alcohol drinkers with positive HPV compared to alcohol drinkers with negative HPV [190]. These discrepancies could be attributed to variations in data collection methods and reporting formats [190].

In summary, alcohol consumption, a recognized carcinogen, synergistically heightens the risk of HPV-induced carcinogenesis, particularly in head and neck cancers and cervical cancer. It induces oxidative stress, DNA damage, and epigenetic modifications, fostering viral infection, persistence, and tumor development. While some studies suggest a significant association between alcohol use and HPV infection, conflicting findings underscore the need for further research to clarify the interaction's complexity and its implications for cancer prevention and management.

### Nutrition and HPV-induced carcinogenesis

In cervical cancer, maintaining a healthy diet can both prevent and counter HPV infection. This is achieved by safeguarding DNA from damage through the action of antioxidant vitamins that neutralize free radicals [191]. Regarding the antioxidant impact of vitamins, ascorbic acid (vitamin C) is a scavenger of free radicals, reducing O2^·^ and OH^·^ as well as lipid hydroperoxides [192]. Vitamin E (tocopherol), a powerful lipid-soluble antioxidant, safeguards immune cell membranes by countering ROS and reducing PUFAs' oxidation. It also indirectly regulates the immune system by inhibiting PGE2 synthesis, potentially aiding in HPV prevention [193]. Riboflavin (vitamin B2) participates in metabolic redox processes, and the metabolism of 1-carbon, and its deficiency can lead to changes in DNA methylation [194]. Vitamin B12 and folic acid hinder HPV genome methylation, reducing viral replication and persistence of infection; however, their deficiency can facilitate HPV DNA integration through DNA fragmentation [195]. Folic acid can also reduce the risk of HPV infection [196]. It has been found that vitamin E exerts its antioxidant function by eliminating ROS and oxidation of unsaturated fatty acids in the membrane of immune cells to prevent DNA damage [197]. Their isoforms and metabolic products also have anti-inflammatory and anti-cancer impacts [198]. Although the exact mechanism of its effect on HPV has not yet been determined, the use of this vitamin in combination with other micronutrients has been reported to be beneficial. In recent years, the relationship between the risk of cervical cancer and the consumption of carotenoids, vitamin E, and folate has been investigated [199]. Letafati et al. reviewed the antioxidant role of vitamins C, A, D, E, B1, B2, and folate, as well as components like Alpha lipoic acid, carotenoids, and the polyphenols of Olive Leaf in antioxidant defense against HPV-induced carcinogenesis and cervical cancer [200]. A study in American women provides the first reports of an connect between serum vitamin C levels and HPV infection. They claim that although there was no relationship between vitamin C serum levels and infection in women under 25 years of age, a U-shaped relationship was seen in older women with HPV. This means that those who had a serum level of 69.5 µmol/L had the lowest risk of infection, and those who had a serum level lower or higher than this amount had a higher risk [201]. Research indicated that antioxidants can reduce the proliferation of the virus by modulating the immune response and thus control the advancement of cervical cancer [199]. The results of a study showed that women who have a higher composite dietary antioxidant index (CDAI) are less prone to HR-HPV infection than others [202]. In a study, researchers investigated the concentration of folate while controlling the consumption of beneficial micronutrients (vitamin E, B12C, and total carotene) in women exposed to cervical neoplasia. High-risk HPV status with the hybrid absorption method 2 (HC-2) was at least three times in women. The results showed that a higher concentration of folate can reduce the possibility of getting high-risk HPV [203]. EGCG is a catechin with antiviral effects that target different stages of virus entry to fusion [204]. In a study, the effect of Epigallocatechin-3-gallate (EGCG), which is a primary bioactive polyphenol in green tea, was investigated on the growth rate and differentiation of keratinocytes transformed by the HPV virus. In this study, it was suggested that EGCG acts as an anti-virus against E6 and E7 oncoproteins; the destruction of these two is done by proteasome in cells. As a result, the expression of p53, p21WAF1, and pRB and the reduction of cell proliferation are especially beneficial. Accumulation of p53, as well as p21WAF1, can inhibit cell proliferation [205]. Another study was conducted to investigate the expression of antiviral IFN-stimulated genes (ISGs) in keratinocytes transfected with HPV-2 in skin warts. Pretreatment (and not posttreatment) with EGCG can hinder HPV-2 E7 transfection. EGCG can probably, maintain the expression of ISGs and IFN-1 signaling cascade components. The impact of pretreatment with EGCG have been reported in many viral diseases, including HIV-1, WNV, and influenza [206]. Savini et al. showed that folate deficiency was linked to elevate in the level of intracellular homocysteine, which leads to the regulation of the level of hnRNP E1 and interferes with the expression of L2, resulting in the absence of complete virus production. The lack and then the replenishment of folate levels in keratinocyte cells immortalized with HPV16 can enhance the carcinogenic effects of HPV through the change of cell phenotype [207]. Analyzing the effect of folate deficiency on the integration of HPV16 in keratinocytes of an animal model, Xiao Suhong and the authors showed that the cells of folate-deficient mouse models express more HPV 16 E6 and E7 than mice with a higher folate diet. Also, in these cells, the integration of the virus gene with genomic DNA directly or in the vicinity of the cell was about twice as high [208]. A study in American women aged 30–45 with persistent HPV infection showed that combined treatment with hyaluronic acid (HA), folic acid, EGCG, and vitamin B12 for 12 weeks reduced the persistence of the virus. This combination showed positive and effective results for the treatment of patients with (low-grade squamous intraepithelial lesion) LSIL and (atypical squamous cells- undetermined significance) ASC-US [209]. In a case report in 2023, a 39-year-old patient with severe intraepithelial neoplasia of the cervix (CIN3), with positive HPV 16 and diagnosis of the need for hysterectomy, was included in a study to investigate the effects of vitamin B12, EGCG, hyaluronic acid, and folic acid instead of surgery or before surgery. After eight weeks, there was no trace of cellular and nuclear atypia and abnormal proliferation. There was only chronic cervicitis without malignant signs. Examinations six months later also showed negative colposcopy, Pap test, and an HPV DNA test, which can confirm the hypothesis of the effectiveness of the mentioned oral compound on internal cervical lesions and HPV status [195]. Table 1 shows the role of dietary antioxidants such as vitamins C, A, E, B12, K, and folate in HPV infection and carcinogenesis. It seems that the effectiveness of all compounds can provide a clear perspective on the prevention of HPV infection and the treatment of wounds, warts, and cancers caused by this virus in the future.

**Table 1:** Role of dietary antioxidants such as vitamin C, A, E, B12, K and folate in HPV infection and carcinogenesis

Vitamin	HPV	Mechanism	Optimal vit intake^a^
Vit C	Decreased HPV infection in women ≥ 25 years [201] Decreased risk of persistent HPV infection [210] Decreased risk of cervical neoplasia (OR = 0.58) [211]	Decreased ROS Enhance the host's cellular and humoral immunity [210] Downregulation of the HPV E6, negatively regulates the redox-sensitive transcription factor AP-1 (c-jun and c-fos) and stabilizes P53 [212] Inhibit the formation of DNA adduct [213]	69.5 µmol/L [201]
Vit A	Decreased cervical cancer risk (OR = 0.35) [213] One log2 unit increase in vitamin A was related to 10% reduction in the risk of HPV infection (when vitamin A is < 1448.155mcg) [214]	Regulation of cell differentiation and proliferation [213]	1448.155 mcg [214]
Vit E	Deceased HR-HPV infection (aOR^b^ = 0.72) [193] Deceased cervical cancer risk (OR = 0.53) [213]	Scavengers of ROS Regulation of cell differentiation and proliferation Enhance immunological function Inhibit DNA adduct formation [213]	> 10.82 mg/day [193]
Folate	With a tertile elevation in the plasma folate was 50% increase in the odds of having HPV 16 - Of HPV + individuals with elevated serum folate, 75% fewer were diagnosed with CIN 2 + [215]	Methylation of HPV DNA on CpG sites of promoter and suppress viral replication [209, 215]	> 6 ng/mL [216]
Vit B12	With a tertile elevation in the plasma vitamin B12, 40% increase in the odds of having HPV 16 Of HPV + individuals with elevated serum B12, 60% fewer were diagnosed with CIN 2 + [215] Safeguarding males from non-oncogenic HPV persistence [217]	Methylation of HPV DNA on CpG sites of promoter and suppress viral replication [209, 215]	> 356 pg/mL [216]
Vit k	In the range of 0–3.81, one unit elevation in log2 vitamin K was related to 43% reduction in the risk of HPV infection related to 43% reduction in the risk of HPV infection [218]	Decreased circulating inflammatory mediators Prevent IkB release from NF-kB to allow its entry to the nucleus [218]	> 14.03 mcg [218]

^a^Optimal dietary vitamin intake significantly reduces the risk of HPV infection

^b^Adjusted OR

### Psychological stress and HPV-induced carcinogenesis

In psychological stress, corticotropin-releasing factor (CRF) regulates the stress response by initiating the hypothalamic–pituitary–adrenal axis, trigger the release of glucocorticoids [219]. Stress hormones, such as glucocorticoids (cortisol and epinephrine), trigger the DNA damage response by generating OS (induction of ROS, RNS) via binding to glucocorticoid receptor (GR) [220, 221]. This process involves the activation of kinases ATM and ATR, which result in cell-cycle arrest, DNA repair, and apoptosis. Glucocorticoids also interfere with DNA repair that is controlled by RAD51 and BRCA1, causing to the buildup of DNA damage [221]. In reaction to psychological stress, cortisol displayed heightened nitric oxide synthase (iNOS) expression, and glucocorticoid receptor-associated Src kinase was related to cortisol-induced production of RNS [220]. Psychological stress can indeed play a role in inducing OS and the production of O_2_^·−^, OH, and H_2_O_2_ in the brain [222]. In a mouse model study, psychological stress-induced oxidative damage disrupted the granulocyte-lymphocyte balance, increasing granulocyte count, elevating ROS production, and raising peroxynitrite levels and lipid peroxidation in the brain, heart, liver, and spleen [223].

Psychological stress can protract HPV's severity and duration through the activation of stress hormones, which may reawaken latent viruses, boost the expression of HPV oncogene, decrease the release of interferon, and hinder the body's antiviral protection [224, 225]. Exposure to HR-HPV to glucocorticoids and catecholamine may increase oncogene expression, interact with host cell proto-oncogenes, and escape detection by the immune system through the suppression of HLA molecule expression [225]. Acute stress can boost peripheral blood lymphocyte levels, particularly NK cells. Short-term stress is linked to changes in cytokine secretion and a shift in immunity response from Th1 toward Th2. The decline in NK cell activity and lymph proliferation shifts the immune response toward Th2 and decreases secretion of IFN-γ and IL-2, resulting in weakened cellular immunity in chronic stress, potentially increasing the risk of infections and cancer [226]. Elevated levels of psychological stress have been accompanying to a compromised immunity response to HPV in women who have dysplasia of the cervix. This diminished response appears to be related to a weakened functionality of T-cells when confronting HPV16. This may occur due to alterations induced by stress in the production of Th1/Th2 cytokines, ultimately fostering viral persistence and facilitating the progression of cancer [227]. Similarly, in a research study, 426 men underwent semi-annual HPV testing, while their stress levels were classified as either high or low based on the Perceived Stress Scale (PSS-4). The prevalence of HR-HPV infection is 1.53 times higher [PR = 1.53 (95% CI: 1.06–2.20)] in the group with high-life stress events. Additionally, high stress is linked to a lower rate of clearing HPV infections in men over 50 years of age [228].

A cohort study conducted on 4.6 million individuals revealed that psychological stress resulting from the loss of a child was connected to an increased risk of HPV-related cancers, including cervical cancer (RR: 1.46; 95% CI: 1.17–1.80) [229]. Kuebler et al. investigated the links between chronic stress, diurnal cortisol patterns, and HPV infection at the beginning and after a one-year follow-up. Higher chronic stress, specifically from work pressure and anxiety, along with a higher cortisol awakening response (CAR), were associated with HR-HPV positivity at the beginning of the test [230]. Additionally, glucocorticoids induce HPV-E6 expression and reduce miR-145 in cervical cancer cells, resulting in p53 dysfunction and promoting chemotherapy resistance. MiR-145 enhances p53's tumor-suppressing effects and inhibits cancer cell motility [231]. In a study involving 1,696 women who had experienced the loss of a family member, this stress was correlated with a 62 percent higher likelihood of contracting HPV-16 infection, along with a heightened viral load and the occurrence of repeat HPV infections. Psychological stress was also linked to a 4–9% heightened risk of initial abnormal cervix cytology [232]. Stressful life also plays a role in the progression of HPV viral infections towards carcinogenesis. A study's findings revealed that women co-infected with HR-HPV and HIV, who also experienced 6 months of stress, had a seven-fold increased risk of developing persistent or progressive squamous intraepithelial lesions (SIL) in the cervix a year later [233]. Additionally, exposure to marital life stress was linked to the occurrence of SIL in the cervix, especially in the presence of HR-HPV [234]. The likelihood of developing cervical cancer was higher in women with high-stress occupations. Apart from the influence of life stress, conditions like depression and bipolar disorder are also associated with cervix cancer in a cohort study [235].

**Fig. 3:**
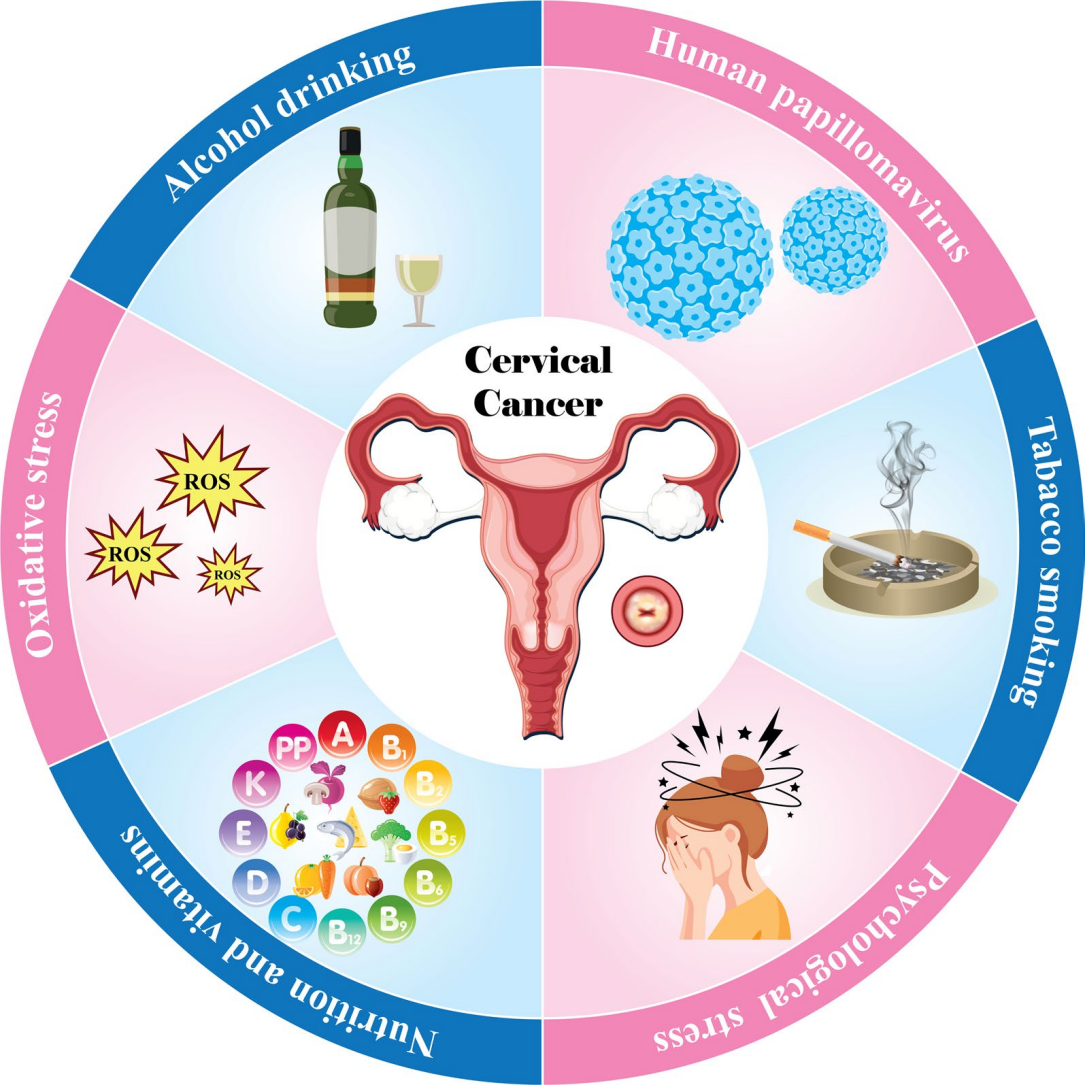
Cervical cancer and oxidative stress factors

In summary, lifestyle choices like diet, alcohol intake, smoking, and psychological stress, as well as genetic and epigenetic changes and viral oncoproteins, can affect OS, influencing the development of HPV-related cancers (Fig. 3). Understanding and controlling OS is crucial for preventing and treating cancer. This study proposes investigating dietary antioxidants and targeting cancer cells using reactive oxygen species mechanisms as potential therapeutic strategies to reduce the impact of OS on HPV-linked malignancies.

## Conclusion

HPV infection is a significant risk factor for cancer, but it operates in conjunction with other complex mechanisms, most notably OS. OS arises from various sources, including lifestyle choices like smoking and alcohol consumption and even psychological stress. OS plays a crucial role in DNA damage and tumorigenesis in HPV-related cancers. HPV promotes carcinogenesis through OS by various mechanisms, such as altering mitochondrial cristae, raising ROS levels and DNA damage by lowering the SOD 2 and GPx 1/2 antioxidant, activating the EGFR/MEK/ERK cell signaling pathway, and upregulating the OS sensor Pirin, and also causing LDHA translocation due to elevated ROS caused by HPV oncoprotein and subsequent methylation of histone H3K79. Furthermore, HPV-infected cells adapt to OS situations by inhibiting OS-induced apoptosis and modulating antioxidant activity via its oncogenes, which bypasses DNA damage response pathways. On a positive note, dietary antioxidants such as vitamins offer a potential defense against HPV infection by neutralizing free radicals and safeguarding DNA. Understanding the relationship between OS, HPV, and cancer is essential for developing strategies to prevent and treat HPV-related malignancies. Current HPV-related cancer treatments involve surgery and chemotherapy, but ongoing research may yield more effective strategies targeting OS for improved outcomes.

## Data Availability

Not applicable.
